# Survivin polymorphisms and susceptibility to prostate cancer: A genetic association study and an *in silico* analysis

**DOI:** 10.17179/excli2018-1234

**Published:** 2018-05-18

**Authors:** Mohammad Karimian, Younes Aftabi, Tahereh Mazoochi, Faezeh Babaei, Tahereh Khamechian, Hossein Boojari, Hossein Nikzad

**Affiliations:** 1 Gametogenesis Research Center, Kashan University of Medical Sciences, Kashan, Iran; 2 Anatomical Sciences Research Center, Kashan University of Medical Sciences, Kashan, Iran; 3 Department of Pathology, Faculty of Medicine, Kashan University of Medical Sciences, Kashan, Iran; 4 Department of Statistics, Faculty of Sciences, Islamic Azad University, Kashan Branch, Kashan, Iran

**Keywords:** prostate cancer, survivin gene, genetic polymorphism, in silico analysis

## Abstract

Survivin is a member of the apoptosis inhibitor protein family and its polymorphisms may lead to susceptibility to cancer. The aim of this study was to investigate the possible association of c.-31G>C (rs9904341), c.454G>A (rs2071214), c.*148T>C (rs2239680) and c.*571T>C (rs1042489) polymorphisms of *survivin* gene with prostate cancer risk and provide some justification using *in silico* analysis. The 157 men with prostate cancer and 145 healthy controls were included in a case-control study. The studied polymorphisms were genotyped using PCR-RFLP method. An *in silico* approach was employed to show the possible effects of the polymorphisms on the survivin gene function. The study revealed that there are significant associations between c.-31CC genotype (OR= 2.29, 95 % CI= 1.20-4.37, *p*= 0.012), c.-31C allele (OR= 1.62, 95 % CI= 1.17-2.26, *p*= 0.004), c.454AG genotype (OR= 2.03, 95 % CI= 1.02-4.04,* p*= 0.043), and c.*148C allele (OR= 1.49, 95 % CI= 1.04-2.15, *p*= 0.031) with prostate cancer. Using stratified analysis, we found also significant effects of age distribution on the association of c.-31G>C with prostate cancer risk (OR= 2.10, 95 % CI= 1.08-4.10, *p*= 0.030). Also as a preliminary study, it was shown that smoking status has significant effects on the association of c.-31G>C (OR= 1.94, 95 % CI= 1.08-3.49, *p*= 0.027) and c.*148T>C (OR= 2.60, 95 % CI= 1.47-4.60, *p*= 0.001) polymorphisms with prostate cancer risk. Finally, *in silico* analysis revealed that c.-31G>C, which is located in a CpG island of the promoter may change transcriptional regulation of *survivin* gene and c.454G>A and *148T>C could affect protein structure and possible miRNA interaction with 3'-UTR of *survivin *transcript respectively. According to the results, c.-31G>C, c.454G>A, and c.*148T>C polymorphisms could be genetic risk factors for prostate cancer in an Iranian population. However, further studies with larger sample size and different ethnicities are required to obtain more comprehensive results.

## Introduction

Prostate cancer is one of the common form of malignancies in men around the world (Schröder and Roobol, 2009[34]). The occurrence of this cancer is increasing because of some exogenous and endogenous factors, including smoking, job-related contacts to chemical compounds and chronic infectious diseases (Grönberg, 2003[12]). Also, genetic factors play an important role in the susceptibility to prostate cancer (Mendiratta and Febbo, 2007[28]). Folate metabolism and apoptosis are of the key pathways, which are involved in the prostate cancer development and progression. Therefore, genetic variations in these pathways may influence the prostate cancer risk in different individuals and populations (Ho et al., 2011[14]).

Survivin is an apoptosis inhibitor and plays a negative regulatory role in this cellular event. It suppresses apoptosis progression by inhibition of the initiator caspase 9 and executioner caspases 3 and 7 (Kotipatruni et al., 2012[22]). Also, survivin acts as an essential regulator of cell division especially in G1 to S transition of the cell cycle. Its expression in many tissues is limited but it is highly expressed in cancer cells, which suggests the direct role of survivin in tumorigenesis (Eslami et al., 2016[9]). 

The *survivin* gene, also called *BIRC5*, locates on chromosome 17 (17q25.3) and its encoded protein contains a BIR (Baculoviral IAP Repeat) domain (Altieri, 2001[2]). Given the role of survivin in the carcinogenesis, so the varieties in this gene should be considered as potential markers for the diagnosis of cancer (Yang et al., 2009[42]; Zhu et al., 2013[43]). There is a common single nucleotide polymorphism (SNP) in the promoter region of *survivin* gene (c.-31G>C, rs9904341), which is located in the CDE/CHR repressor element and may result in the overexpression. Evidences obtained from previous examines have shown that this polymorphism is associated with a variety of cancers such as colorectal and gastric cancers (Qin et al., 2014[32]), and nasopharyngeal carcinoma (Chen et al., 2013[6]). Also, it is reported that the SNP have an effect on age of onset of ovarian cancer (Han et al., 2009[13]). Moreover, there are two functional SNPs in the 3'-UTR of *survivin* (c.*571T>C, rs1042489; and c.*148T>C, rs2239680), which may alter post-transcriptional regulation of the gene (Shi et al., 2012[35]). It's reported that carriers of the minor allele of c.*571T>C among breast cancer patients have a worse survival compared with the major homozygotes. Also, it is shown that c.*148T>C may increase individual susceptibility to lung cancer probably by attenuating the interaction between miR-335 and survivin mRNA (Zu et al., 2013[44]). Further, there is a missense transition, c.454G>A (rs2071214), on exon 6 of *survivin,* which results in p.Glu152Lys substitution and may affect protein structure and function. A meta-analysis claimed that this SNP seemed to be associated with an increased tumor risk in Asians (Zhu et al., 2013[43]) and another study reported its association with familial breast cancer risk (Kabisch et al., 2015[18]). In this study, at first we investigated the association of *survivin* gene polymorphisms c.-31G>C, c.454G>A, c.*148T>C and c.*571T>C with prostate cancer and then, an *in silico* analysis was done to provide possible justification for the association results.

## Materials and Methods

### Subjects

In a case-control study, 157 patients with prostate cancer and 145 age-matched healthy controls were randomly included. Case subjects were recruited from prostate cancer patients admitted to oncology department of Shahid Beheshti hospital (Kashan, Iran) between 2014 and 2015. Prostate cancer was confirmed by elevated PSA serum levels (>2.5 ng/ml), digital rectal examination (DRE), and histopathology results. The Gleason score of patients was evaluated by a pathologist using the Gleason scoring system. Patients with other malignancies were excluded from this study. The individuals referring to the hospital for routine check-up examination who had PSA levels <2.5 ng/ml and/or normal DREs were included as control group. The subjects with symptoms and signs of any malignancy and family history of cancer were excluded from the control group. After obtaining signed informed consent, 2 ml blood was taken from all subjects and preserved in CBC tubes at -20^°^ C for further usages. Research protocols were approved by medical ethic committee of research council of Kashan University of Medical Sciences at Aug. 2014 (Ref no. IR.KAUMS.REC.1395.92).

### DNA extraction and SNPs genotyping

Genomic DNA was isolated from peripheral blood samples using salting-out procedure. The genotypes of c.-31G>C, c.454G>A, c.*148T>C and c.*571T>C polymorphisms of *survivin* gene were determined by PCR-RFLP method. Primers around the SNPs were designed by utilizing Oligo7 software. The specific primers sequences are listed in Table 1(Tab. 1). PCR was carried out in a total volume of 25 µl consisting of 2.5 µl of 10X PCR buffer, 0.35 µM each of the sense and antisense primers, 0.75 µl of 50 mM dNTPs mixture, 2 units of *Taq* DNA polymerase, and 50 ng of genomic DNA (all of PCR components were purchased from CinnaGen Co., Tehran, Iran). The PCR was done in a peqSTAR thermal cycler system (PeqLab, Erlangen, Germany) using the following conditions: initial denaturation at 94°C for 5 min, followed by 35 repetitive cycles of denaturation at 9 °C for 45 sec, annealing at 60 °C (for c.-31G>C), 57 °C (for c.454G>A), 58 °C (for c.*148T>C) and 56 °C (for c.^*^571T>C) for 45 sec, and polymerization at 72 °C for 40 sec, and a final polymerization at 72 °C for 7 min. PCR products of the SNPs c.-31G>C, c.454G>A, c.*148T>C and c.*571T>C were treated by *EcoO109*I*, Sac*II*, Ava*II*, Msp*I restriction enzymes, respectively. After incubation of *EcoO109*I and *Msp*I enzymatic mixtures at 37 °C for 16 hours, they were electrophoresed onto 1 % agarose gels and visualized by GreenView safe staining (Applied BioProbes Co., USA). But, *Sac*II and* Ava*II enzymatic mixtures were electrophoresed onto 8 % polyacrylamide gels and visualized by silver nitrate (AgNO_3_) staining. About c.-31G>C polymorphism, the digested samples showed three different patterns: genotype GG, with 269- and 126-bp fragments, genotype CC with 395-bp fragment and genotype GC with 395-, 269-, and 126-bp fragments; given to c.454G>A transition: genotype GG with 100- and 22-bp fragments, genotype AA with 100-bp fragment, and genotype AG with 122-, 100-, and 22-bp fragments; concerning c.*148T>C polymorphism: genotype CC with 121- and 21-bp fragments, genotype TT with 142-bp fragment, and genotype TC with 142-, 121-, and 21-bp fragments. With regard to c.^*^571T>C polymorphism, genotype CC, with 297- and 179-bp fragments, genotype TT with 476-bp fragment and genotype CT with 476-, 297-, and 179-bp fragments. Finally, DNA direct sequencing was used to approve the PCR-RFPL procedures. For this purpose, one sample from each genotype was sequenced in Bioneer Co. (Korea) using an automated DNA-sequencing. 

### In silico analysis

F-SNP database (http://compbio.cs.queensu.ca/F-SNP/), which provides valuable information about the effects of SNPs (Lee and Shatkay, 2007[24]) was used for discovering the possible effects of the c.-31G>C SNP on *survivin*-gene function. Also, PNImodeler server (http://165.246.44.34/pnimodeler/) that predicts protein-binding sites in a DNA sequence (Im et al., 2015[15]) was used to evaluate F-SNP database report and to determine possible effects of c.-31G>C SNP on protein binding sites in *survivin* promoter sequence. Considering that G to C transversion in promoter region may affect methylation statues of a CpG island in a regulatory sequence we used DataBase of CpG islands and Analytical Tool: DBCAT (Kuo et al., 2011[23]) to identify if -31G>C locates in a CpG island (http://dbcat.cgm.ntu.edu.tw/). Also, the effects of c.454G>A exonic polymorphism on the structure and function of protein were evaluated by some bioinformatics tools. For example, hydrophobicity and average flexibility of the protein was evaluated by ExPASy web server. Effect of c.454G>A polymorphism on secondary structure was evaluated by SOPMA secondary structure prediction method (Sapay et al., 2006[33]). The SNAP software was used to evaluate the overall effect of c.454G>A polymorphism on the function of survivin (Bromberg and Rost, 2007[5]). The miRNA SNP ver2.0 database was employed for assessment of miRNA interaction with 3'-UTR of *survivin *mRNA after c.*148T>C substitution (Gong et al., 2012[11]). 

### Statistical analysis 

An independent t-test was used for analysis of numerical variables. Hardy-Weinberg equilibrium (HWE) was calculated for both case and control groups. A binary logistic regression was used to estimate odd ratios (ORs) with a 95 % confidence interval (CI). Differences in the frequencies of alleles and genotypes between the case and control groups were assessed by a χ^2^ test. The *p*-values less than 0.05 were considered as statistically significant. All of these statistical analyses were performed by the SPSS version 19 statistical software package (SPSS, Inc, Chicago, Illinois).

## Results

### Characteristics of the study population

Some clinical and demographic details of study subjects are presented in Table 2(Tab. 2). There were no statistically significant differences for age, body mass index (BMI), and status of smoking between cases (mean age of 64.90 ± 12.48, mean BMI of 23.25 ± 2.77, and 62.42 % of ever smoking) and controls (mean age of 66.69 ± 7.77, mean BMI of 23.51 ± 2.58, and 71.03 % of ever smoking). Additionally, PSA level, and Gleason score were listed in Table 2(Tab. 2).

### Associations of survivin gene polymorphisms with prostate cancer 

The allele and genotype frequencies of c.-31G>C, c.454G>A, c.*148T>C, and c.*571T>C polymorphisms are summarized in Table 3(Tab. 3). In the case of c.-31G>C polymorphism, the frequencies of GG, GC, and CC genotypes in control group are 47.59 %, 37.93 %, and 14.48 %, respectively while these percentages in case group are 33.76 %, 42.67 %, and 23.57 %, respectively. Statistical analysis revealed that GC genotype does not increase the risk of prostate cancer (OR: 1.59, % CI= 0.96-2.63, *p*= 0.074). But, there was a significant association between homozygous CC and prostate cancer in our study population (OR= 2.29, 95 % CI= 1.20-4.37, *p*= 0.012). Also, carriers of C allele (GC+CC) were at a high risk for prostate cancer (OR= 1.78, 95 % CI= 1.12-2.83, *p*= 0.015). Allele analysis revealed that C allele is a risk factor for prostate cancer (OR: 1.62, % CI= 1.17-2.26, *p*= 0.004). Concerning c.454G>A transition, heterozygote (AG) genotype was associated with risk of prostate cancer (OR= 2.03, 95 % CI= 1.02-4.04, *p*= 0.043). Furthermore, there was a significant association between carriers of G allele (AG+GG) and prostate cancer risk (OR= 1.96, 95 % CI= 1.01-3.84, *p*= 0.048). Given to c.*148T>C transition, we found that carriers of C allele (TC+CC) were at a high risk for prostate cancer (OR= 1.62, 95 % CI= 1.03-2.56, *p*= 0.037). Also there was a significant association between C allele and prostate cancer risk (OR= 1.49, 95 % CI= 1.04-2.15, *p*= 0.031). With regard to the c.*571T>C transition, there was no significant association of TC (OR= 1.27, 95 % CI= 0.77-2.10, *p*= 0.339) and CC (OR= 1.50, 95 % CI= 0.66-3.42, *p*= 0.330) genotypes with prostate cancer risk. In addition, we found no significant association between c.*571T>C transition and prostate cancer in C *vs*. T (OR= 1.28, 95 % CI= 0.89-1.86, *p*= 0.186) and TC+CC *vs*. TT (OR= 1.32, 95 % CI= 0.83-2.10, *p*= 0.236) genetic models.

### Stratified analysis

As a preliminary study, the associations of the four *survivin* gene polymorphisms with risk of prostate cancer were assessed by stratified analysis via age, BMI, and smoking status. When the c.-31G>C polymorphism in combination with age was studied in relation to prostate cancer risk, a significant association was observed in a dominant model (Table 4(Tab. 4)). When GG genotype with age less than 69 years was considered as reference, carriers of C allele who were younger than 65 years showed a significant increased risk for prostate cancer (OR= 2.10, 95 % CI= 1.08-4.10, *p*= 0.030). Moreover, after stratifying of analysis by smoking status, we found that there were significant associations between c.-31G>C (OR= 1.94, 95 % CI= 1.08-3.49, *p*= 0.027) and c.*148T>C (OR= 2.60, 95 % CI= 1.47-4.60, *p*= 0.001) polymorphisms and prostate cancer in smoker subjects (Table 4(Tab. 4)). Additionally, no significant associations were observed between the c.454G>A and c.*571T>C and risk of prostate cancer in the stratified analysis (data not shown).

### In silico analysis

**Figure 1:**
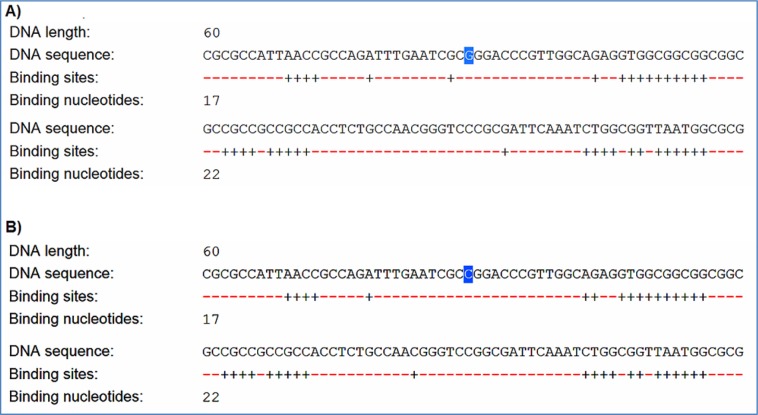
PNImodeler server prediction. (+) and (-) signs represent probable binding and nonbinding site on DNA strand respectively. A) Depicts probable nucleotides that may interact with protein around G allele of rs9904341 both in forward and revers sequences of promoter. B) Represents probable protein binding site in promoter sequence when there is a C nucleotide in -31 position.

**Figure 2:**
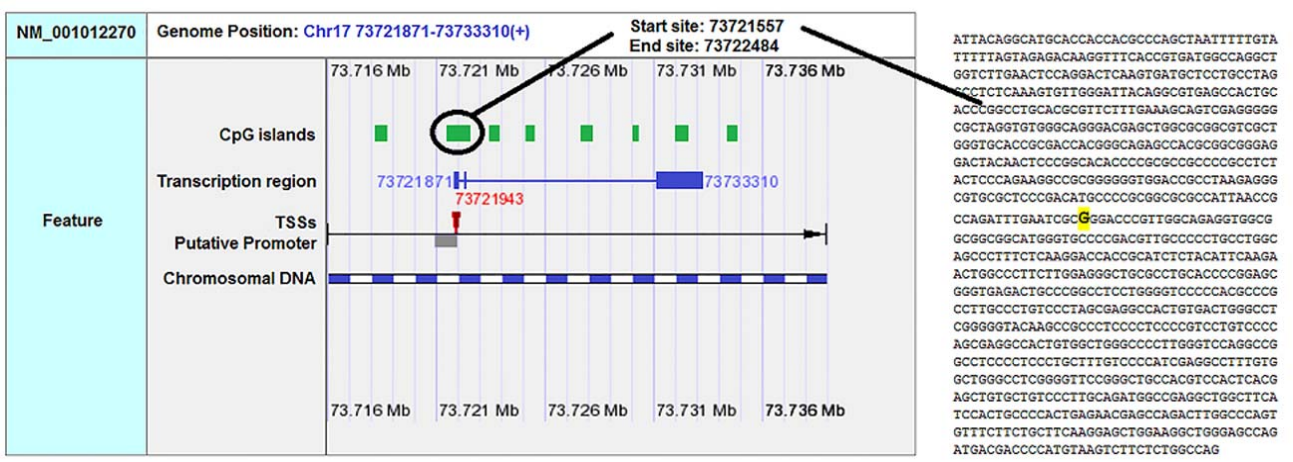
DBCAT predicts CpG islands of *survivin* gene *BIRC5*. -31G>C transversion that is highlighted in the right section of this figure locates in a CpG island.

**Figure 3:**
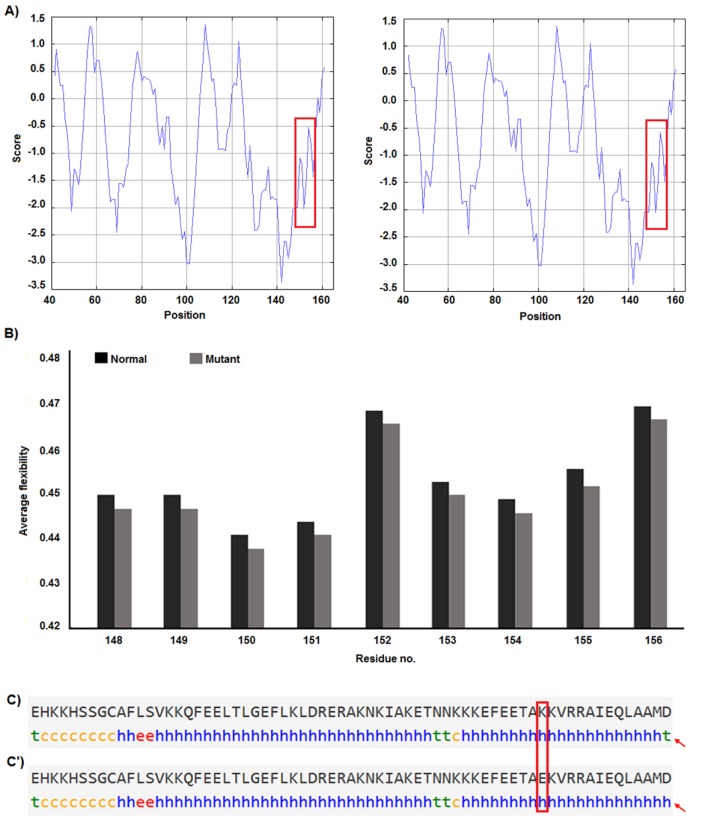
Hydrophobicity, average flexibility and secondary structure of survivin after c.454G>A transition. The hydrophobicity (A) and average flexibility (B) of protein alter in residues 148 to 156. Secondary structure of alters at the c-terminal of the protein (C&C').

F-SNP showed that rs9904341-SNP may change transcriptional regulation of *survivin* gene (Table 5(Tab. 5)). PNImodeler server predicted that the rs9904341-SNP alters binding nucleotides around SNP in both forward and revers strands of promoter DNA (Figure 1(Fig. 1)). DBCAT showed that in *survivin* gene *BIRC5*, there is a CpG island, which starts from 73721557 and ends in 73722484 nucleotide (Figure 2(Fig. 2)). The SNP position in the sequence is 73721963 and it occurs in *BIRC5*-gene CpG Island. With regard to c.454G>A SNP, we found that this polymorphism results in lysine to glutamate substitution at codon 152 (Glu152Lys). Bioinformatics data revealed that this substitution reduces hydrophobicity and average flexibility of the protein at residues 148 to 156 (Figure 3(Fig. 3)). Also, it could generate a minor change in the secondary structure at the C-terminal of protein (Figure 3(Fig. 3)). Moreover, the data from SNAP web server revealed that Glu152Lys substitution could be damaging for protein function (Score= 7; expected accuracy= 53 %). Finally, we evaluated the effects of c.*148T>C transition on miRNA interaction with 3'-UTR of *survivin *mRNA by miRNA SNP ver2.0 server. Our data revealed that this substitution reduces the interaction of has-mir-335 with 3'-UTR of *survivin *transcript. This SNP could alter the interaction energy between has-mir-335 and 3'-UTR from -19.30 to 0.0 kcal/mol (Table 6(Tab. 6)).

## Discussion

In this study, we investigated the association of four common polymorphisms of *survivin* gene (c.-31G>C, c.454G>A, c.*148T>C and c.*571T>C) with prostate cancer which followed by a bioinformatics analysis to provide possible justification for association results. The experiments revealed that there are significant associations between c.-31G>C, c.454G>A, and c.*148T>C polymorphisms and prostate cancer in the studied population. But, we did not find any significant association between c.^*^571T>C transition and prostate cancer frequency. According to our knowledge, this study is the second report which evaluates the association of survivin gene polymorphisms with prostate cancer risk (Chen et al., 2013[6]). In addition, Chen et al. (2013[6]) reported only the association of -31G/C variant with prostate cancer in Chinese people. While we evaluated the association of four SNPs in survivin gene with prostate cancer risk in Iranian population. Also, some epidemiological studies have been investigating the association of *survivin* gene c.-31G>C polymorphism with the other urinary tract cancers risk. For example, Kawata et al. (2011[20]) and Jaiswal et al. (2012[16]) reported that c.-31G>C transversion is associated with bladder cancer in Japanese and Indian populations, respectively. In addition, Qin et al. (2012[31]) reported that this polymorphism is associated with renal cell cancer in Chinese population while Marques et al. (2013[25]) reported that this polymorphism is not associated with renal cell cancer in southern European population. The different results between these studies may arise from difference in cancer type or ethnicity. In the stratified analysis, we found significant effects of age distribution on the association of c.-31G>C and prostate cancer risk. Also, there were significant effects of smoking status on the association of c.-31G>C and c.*571T>C polymorphisms with the cancer risk. These results show possible interactions among age and smoking status in the etiology of prostate cancer.

Numerous genetic association studies have recognized many susceptibility variants, suggesting the main role of genetic factors in development of prostate cancer (Wiklund, 2010[40]). Then, considering the survivin gene mode of function and expression, it is not surprising searching about the association of its variations with cancer frequency. Indeed, survivin is a tumor specific molecule, which inhibits caspase-9 activation and causes prevention of apoptosis. Also, it has a role in tumor-related angiogenesis (Eslami et al., 2016[9]). The expression of *survivin* gene is elevated in embryonic tissues, whereas its expression is undetectable in differentiated tissues. However, this gene is overexpressed in several tumors (Altieri, 2008[1]) and there is a positive association between the *survivin* overexpression and tumors grade (Duffy et al., 2007[7]). Indeed, according to the evidences, *survivin* could be one of the important diagnostic and prognostic biomarkers for monitoring of tumor progressions (Ghadersohi et al., 2011[10]).

Single nucleotide polymorphism could change the gene expression pattern, mRNA structure and protein function (Ebrahimi et al., 2017[8]; Karimian and Hosseinzadeh Colagar, 2018[19]; Teimouri et al., 2018[37]). Numerous evidences suggested that functional genetic polymorphisms could alter the *survivin* gene expression (Ambrosini et al., 1997[3]). The *survivin* overexpression induced from functional SNPs may result in reduced apoptotic capacity and increased tumor susceptibility (Qin et al., 2014[32]). The c.-31G>C transversion can disrupt the binding site of CDE/CHR repressor and subsequently increase the expression of *survivin* (Xu et al., 2004[41]). Also, *in vitro* analysis revealed that c.-31C allele is more active transcriptionally rather than c.-31G allele. Therefore, individuals with c.-31CC genotype may have up-regulated levels of *survivin* gene (Jang et al., 2008[17]). In addition, c.*148T>C is a key SNP, which could increase the expression of *survivin *in tumor tissues by changing the affinity of miRNA with 3'-UTR of the transcript (Zu et al., 2013[44]).

Some recent publications showed that using *in silico* analysis could be a helpful approach to understand and interpret the polymorphism effect more specifically (Mazaheri et al., 2017[27]; Soleimani et al., 2017[36]). Here we provide an *in silico* approach to approve our experimental study as a novel part of our study. We utilized bioinformatics servers to predict the consequences of c.-31G>C, c.454G>A, and c.*148T>C SNPs in *survivin* gene function. F-SNP uses TFSearch and ConSite to predict the effects of SNPs on TFBS and UCSC Golden Path and Ensembl to retrieve annotated potential regulatory regions like CpG islands. The FS score for c.-31G>C was 0.268 and in this server higher score is assigned to already known disease-related SNPs than to neutral SNPs. F-SNP reported that the c.-31G>C transversion causes to a transcriptional regulation change. Also, PNImodeler prediction revealed that -31G>C transversion changes pattern of probable protein binding sites in *survivin* promoter sequence around transversioned nucleotide that may alter transcription factor interactions with the promoter region and affects gene expression. As is depicted in the Figure 1(Fig. 1), + sites that represent probable binding site on DNA strand is changed both in forward and revers strands of DNA. These events may affect gene expression since of alteration in transcription factor interaction with promoter sequences. In addition, c.-31G>C SNP occurs in a CpG island (Figure 2(Fig. 2)) and any alteration in this sequence may affect methylation status of the promoter. Methylation of CpG Islands has been widely described as a mechanism associated with gene expression regulation (Moarii et al., 2015[29]) especially in prostate cancer (Massie et al., 2017[26]). Also, SNPs that alter methylation pattern of promoter have been reported as important factor in gene expression differences between cells and tissues (Bell et al., 2011[4]) and it is reported that prostate cancer is influenced from such SNP types (Kloth et al., 2012[21]). Also, we evaluated the effects of c.454G>A polymorphism on the structure of protein by *in silico* approach. We observed that some properties of the protein such as hydrophobicity, average flexibility, and secondary structure of protein changed after c.454G>A transition. These changes could alter folding and function of protein (Nicholls et al., 1991[30]; Teng et al., 2010[38]). Then, we assessed the effects of c.*148T>C substitution on the miRNA interaction with 3'-UTR of *survivin *mRNA. We found that this substitution could reduce the interaction of has-mir-335 with 3'-UTR of *survivin *mRNA. Therefore, it may result in *survivin *overexpression and subsequently tumorigenesis (Zu et al., 2013[44]).

Since, the estimation of sample size based on some previous studies investigating the association of survivin gene polymorphisms with urinary system cancers (Wang et al., 2009[39]; Jaiswal et al., 2012[16]) revealed that our sample size is fairly adequate. For example, based on sample size of Wang et al. (2009[39]) study, we estimated the sample size equal to 144 subjects when α value and power considered as 0.05 and 0.8, respectively. But in the stratified analysis, we acknowledge the small sample size issue. Therefore, we considered our stratified analysis as a preliminary study. In addition, we estimated the optimized sample size of our study according to genotype frequencies with α value= 0.05 and power= 0.8 and we found that a sample size equal to 500 subjects (including 250 cases and 250 controls) is enough for this genetic association study.

In conclusion, c.-31G>C, c.454G>A, and c.*148T>C polymorphisms may be risk factors for prostate cancer susceptibility in an Iranian population. But, further studies with larger sample size (about 500 subjects) are required to achieve more accurate results.

There are some limitations in this study which should be considered. Firstly, our small sample size is a great limitation of our study. Also, we did not evaluate the gene-gene interactions in the case-control study. The current study is based on the identification method of the 'one-step-clustering'. This approach has been reported that it might tend to be 'passenger signals' instead of 'drivers', bury the 'real' cancer gene and ignore the interaction of gene-gene, which made the results less robust and accurate. Moreover, we did not evaluate the effects of functional SNPs of *survivin* by *in vitro* approach.

## Notes

Tahereh Mazoochi and Hossein Nikzad (Gametogenesis Research Center, Kashan University of Medical Sciences, Kashan, Iran; E-mail: hnikzad10@gmail.co) contributed equally as corresponding authors.

## Conflict of interest

The authors declare that there is no conflict of interest regarding the publication of this paper.

## Funding

This study was supported by Grants from the Kashan University of Medical Sciences (No. 95089).

## Figures and Tables

**Table 1:**
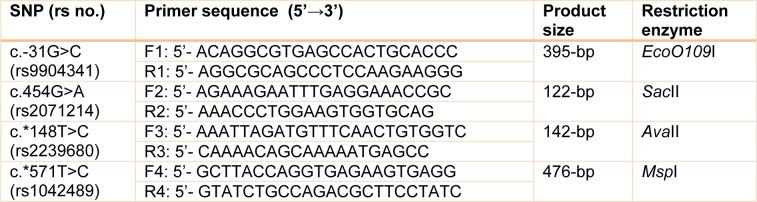
Primer sequences and polymerase chain reaction conditions

**Table 2:**
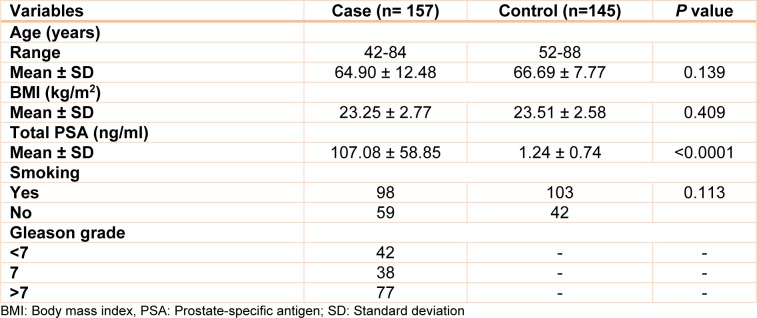
Clinical and demographic details of study subjects

**Table 3:**
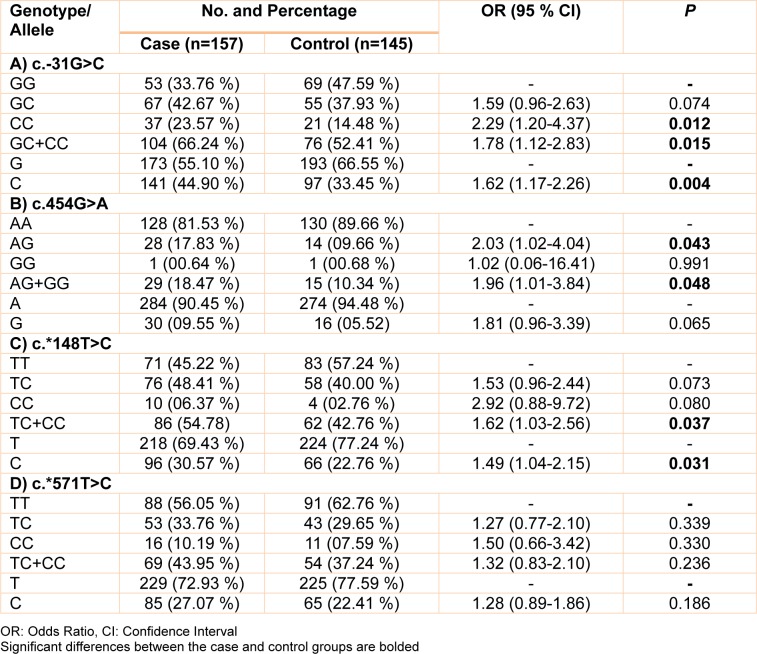
Genotype and allele frequencies of c.-31G>C, c.454G>A, c.*148T>C and c.*571T>C polymorphisms

**Table 4:**
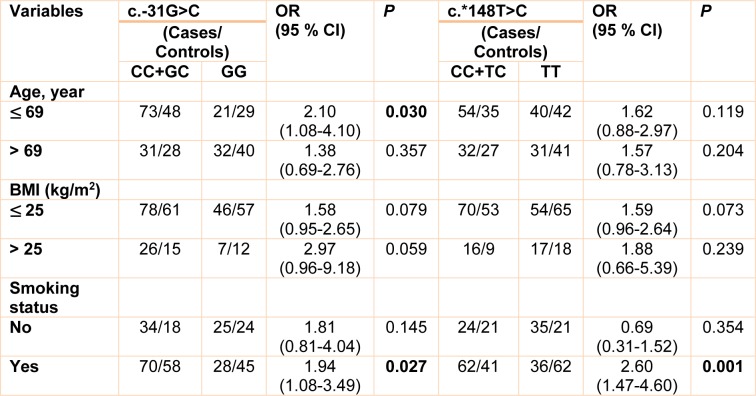
Stratified association analysis between survivin gene polymorphism and prostate cancer risk

**Table 5:**
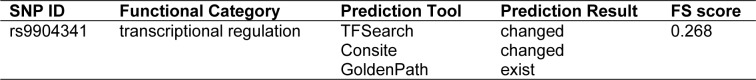
F-SNP results

**Table 6:**
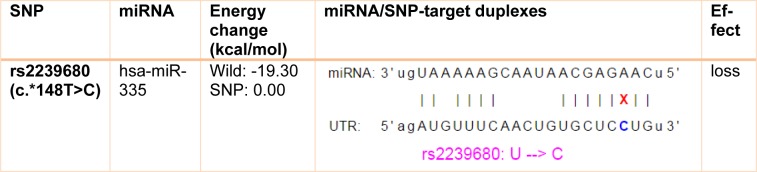
Results of miRNA SNP ver2.0 database

## References

[1] Altieri DC (2008). Survivin, cancer networks and pathway-directed drug discovery. Nat Rev Cancer.

[2] Altieri DC (2001). The molecular basis and potential role of survivin in cancer diagnosis and therapy. Trends Mol Med.

[3] Ambrosini G, Adida C, Altieri DC (1997). A novel anti-apoptosis gene, survivin, expressed in cancer and lymphoma. Nat Med.

[4] Bell JT, Pai AA, Pickrell JK, Gaffney DJ, Pique-Regi R, Degner JF (2011). DNA methylation patterns associate with genetic and gene expression variation in HapMap cell lines. Genome Biol.

[5] Bromberg Y, Rost B (2007). SNAP: predict effect of non-synonymous polymorphisms on function. Nucleic Acids Res.

[6] Chen J, Cui X, Zhou H, Qin C, Cao Q, Ju X (2013). Functional promoter-31G/C variant of Survivin gene predict prostate cancer susceptibility among Chinese: a case control study. BMC Cancer.

[7] Duffy MJ, O'Donovan N, Brennan DJ, Gallagher WM, Ryan BM (2007). Survivin: a promising tumor biomarker. Cancer Lett.

[8] Ebrahimi A, Hosseinzadeh Colagar A, Karimian M (2017). Association of human methionine synthase-A2756G transition with prostate cancer: a case-control study and in silico analysis. Acta Med Iran.

[9] Eslami M, Khamechian T, Mazoochi T, Ehteram H, Sehat M, Alizargar J (2016). Evaluation of survivin expression in prostate specimens of patients with prostate adenocarcinoma and benign prostate hyperplasia underwent transurethral resection of the prostate or prostatectomy. SpringerPlus.

[10] Ghadersohi A, Sharma S, Zhang S, Azrak RG, Wilding GE, Manjili MH (2011). Prostate‐derived Ets transcription factor (PDEF) is a potential prognostic marker in patients with prostate cancer. Prostate.

[11] Gong J, Tong Y, Zhang HM, Wang K, Hu T, Shan G (2012). Genome‐wide identification of SNPs in microRNA genes and the SNP effects on microRNA target binding and biogenesis. Hum Mutat.

[12] Grönberg H (2003). Prostate cancer epidemiology. Lancet.

[13] Han CH, Wei Q, Lu KK, Liu Z, Mills GB, Wang LE (2009). Polymorphisms in the survivin promoter are associated with age of onset of ovarian cancer. Int J Clin Exp Med.

[14] Ho E, Beaver LM, Williams DE, Dashwood RH (2011). Dietary factors and epigenetic regulation for prostate cancer prevention. Adv Nutr.

[15] Im J, Tuvshinjargal N, Park B, Lee W, Huang DS (2015). PNImodeler: web server for inferring protein-binding nucleotides from sequence data. BMC Genomics.

[16] Jaiswal PK, Goel A, Mandhani A, Mittal RD (2012). Functional polymorphisms in promoter survivin gene and its association with susceptibility to bladder cancer in North Indian cohort. Mol Biol Rep.

[17] Jang JS, Kim KM, Kang KH, Choi JE, Lee WK, Kim CH (2008). Polymorphisms in the survivin gene and the risk of lung cancer. Lung Cancer.

[18] Kabisch M, Lorenzo Bermejo J, Dünnebier T, Ying S, Michailidou K, Bolla MK (2015). Inherited variants in the inner centromere protein (INCENP) gene of the chromosomal passenger complex contribute to the susceptibility of ER-negative breast cancer. Carcinogenesis.

[19] Karimian M, Hosseinzadeh Colagar A (2018). Human MTHFR-G1793A transition may be a protective mutation against male infertility: a genetic association study and in silico analysis. Hum Fertil.

[20] Kawata N, Tsuchiya N, Horikawa Y, Inoue T, Tsuruta H, Maita S (2011). Two survivin polymorphisms are cooperatively associated with bladder cancer susceptibility. Int J Cancer.

[21] Kloth M, Goering W, Ribarska T, Arsov C, Sorensen KD, Schulz WA (2012). The SNP rs6441224 influences transcriptional activity and prognostically relevant hypermethylation of RARRES1 in prostate cancer. Int J Cancer.

[22] Kotipatruni RR, Nalla AK, Asuthkar S, Gondi CS, Dinh DH, Rao JS (2012). Apoptosis induced by knockdown of uPAR and MMP-9 is mediated by inactivation of EGFR/STAT3 signaling in medulloblastoma. PLoS One.

[23] Kuo HC, Lin PY, Chung TC, Chao CM, Lai LC, Tsai MH (2011). DBCAT: database of CpG islands and analytical tools for identifying comprehensive methylation profiles in cancer cells. J Comput Biol.

[24] Lee PH, Shatkay H (2007). F-SNP: computationally predicted functional SNPs for disease association studies. Nucleic Acids Res.

[25] Marques I, Teixeira AL, Ferreira M, Assis J, Lobo F, Maurício J (2013). Influence of survivin (BIRC5) and caspase-9 (CASP9) functional polymorphisms in renal cell carcinoma development: a study in a southern European population. Mol Biol Rep.

[26] Massie CE, Mills IG, Lynch AG (2017). The importance of DNA methylation in prostate cancer development. J Steroid Biochem Mol Biol.

[27] Mazaheri M, Karimian M, Behjati M, Raygan F, Hosseinzadeh Colagar A (2017). Association analysis of rs1049255 and rs4673 transitions in p22phox gene with coronary artery disease: A case-control study and a computational analysis. Ir J Med Sci.

[28] Mendiratta P, Febbo PG (2007). Genomic signatures associated with the development, progression, and outcome of prostate cancer. Mol Diagn Ther.

[29] Moarii M, Boeva V, Vert JP, Reyal F (2015). Changes in correlation between promoter methylation and gene expression in cancer. BMC Genomics.

[30] Nicholls A, Sharp KA, Honig B (1991). Protein folding and association: insights from the interfacial and thermodynamic properties of hydrocarbons. Proteins.

[31] Qin C, Cao Q, Li P, Ju X, Wang M, Chen J (2012). Functional promoter-31G> C variant in survivin gene is associated with risk and progression of renal cell cancer in a Chinese population. PloS One.

[32] Qin Q, Zhang C, Zhu H, Yang X, Xu L, Liu J (2014). Association between survivin-31G> C polymorphism and cancer risk: meta-analysis of 29 studies. J Cancer Res Clin Oncol.

[33] Sapay N, Guermeur Y, Deléage G (2006). Prediction of amphipathic in-plane membrane anchors in monotopic proteins using a SVM classifier. BMC Bioinformatics.

[34] Schröder FH, Roobol MJ (2009). Defining the optimal prostate-specific antigen threshold for the diagnosis of prostate cancer. Curr Opin Urol.

[35] Shi H, Bevier M, Johansson R, Enquist-Olsson K, Henriksson R, Hemminki K (2012). Prognostic impact of polymorphisms in the MYBL2 interacting genes in breast cancer. Breast Cancer Res Treat.

[36] Soleimani Z, Kheirkhah D, Sharif MR, Sharif A, Karimian M, Aftabi Y (2017). Association of CCND1 Gene c. 870G> A Polymorphism with Breast Cancer Risk: A Case-Control Study and a Meta-Analysis. Pathol Oncol Res.

[37] Teimouri M, Najaran H, Hosseinzadeh A, Mazoochi T (2018). Association between two common transitions of H2BFWT gene and male infertility: a case-control, meta, and structural analysis. Andrology.

[38] Teng S, Srivastava AK, Wang L (2010). Sequence feature-based prediction of protein stability changes upon amino acid substitutions. BMC Genomics.

[39] Wang YH, Chiou HY, Lin CT, Hsieh HY, Wu CC, Hsu CD (2009). Association between survivin gene promoter− 31 C/G polymorphism and urothelial carcinoma risk in Taiwanese population. Urology.

[40] Wiklund F (2010). Prostate cancer genomics: can we distinguish between indolent and fatal disease using genetic markers?. Genome Med.

[41] Xu Y, Fang F, Ludewig G, Jones G, Jones D (2004). A mutation found in the promoter region of the human survivin gene is correlated to overexpression of survivin in cancer cells. DNA Cell Biol.

[42] Yang L, Zhu H, Zhou B, Gu H, Yan H, Tang N (2009). The association between the survivin C-31G polymorphism and gastric cancer risk in a Chinese population. Dig Dis Sci.

[43] Zhu Y, Li Y, Zhu S, Tang R, Liu Y, Li J (2013). Association of survivin polymorphisms with tumor susceptibility: a meta-analysis. PloS One.

[44] Zu Y, Ban J, Xia Z, Wang J, Cai Y, Ping W (2013). Genetic variation in a miR-335 binding site in BIRC5 alters susceptibility to lung cancer in Chinese Han populations. Biochem Biophys Res Commun.

